# The Role of Impulsivity, Inattention and Comorbid ADHD in Patients with Bulimia Nervosa

**DOI:** 10.1371/journal.pone.0063891

**Published:** 2013-05-20

**Authors:** Jochen Seitz, Berrak Kahraman-Lanzerath, Tanja Legenbauer, Lea Sarrar, Stephan Herpertz, Harriet Salbach-Andrae, Kerstin Konrad, Beate Herpertz-Dahlmann

**Affiliations:** 1 Department of Child and Adolescent Psychiatry, Psychotherapy and Psychosomatics, University Hospital, Aachen, Germany; 2 Clinic for Child and Adolescent Psychiatry, Westphalia-Lippe Regional Association (LWL)-University Hospital Hamm of the Ruhr-University Bochum, Hamm, Germany; 3 Clinic for Audiology and Phoniatry, Charite University Hospital, Berlin, Germany; 4 Clinic for Psychosomatic Medicine and Psychotherapy, Westphalia-Lippe Regional Association (LWL)-University Hospital, Ruhr-University, Bochum Germany; 5 Clinic for Child and Adolescent Psychiatry, Psychotherapy and Psychosomatics, Charite University Hospital, Berlin, Germany; University of Wuerzburg, Germany

## Abstract

**Introduction:**

Little is known about the contribution of impulsivity, inattention and comorbid attention deficit/hyperactivity disorder (ADHD) in the development and maintenance of bulimia nervosa (BN). In particular, their specific contribution to disordered eating symptoms and whether they have additive effects to the general psychopathological burden remains unclear.

**Methods:**

Fifty-seven female patients seeking treatment for BN and 40 healthy controls completed diagnostic questionnaires and interviews that investigated: a) ADHD, b) impulsivity, c) eating disorders and d) general psychopathology. Attentional processes and impulsivity were assessed by a comprehensive computer-based neuropsychological battery.

**Results:**

Twenty-one percent of patients with BN met the clinical cut-off for previous childhood ADHD compared to 2.5% of healthy controls. Adult ADHD according to DSM IV was also more prevalent in patients with BN, with an odds ratio of 4.2. Patients with BN and previous childhood ADHD were more impulsive and inattentive than patients with BN alone. These patients also displayed more severely disordered eating patterns and more general psychopathological symptoms compared with those without ADHD. Severity of eating disorder symptoms was better explained by inattentiveness than by either impulsivity or hyperactivity.

**Discussion:**

Our data suggest an elevated rate of former childhood and current ADHD-symptoms in treatment-seeking patients with BN. Stronger impulsivity and inattention associated with more severe neuropsychological deficits and eating disorder symptoms indicate an additive risk that is clinically relevant for these patients. Thus, clinicians should identify comorbid patients who might profit from additional ADHD-specific treatments.

## Introduction

Recent evidence suggests that bulimia nervosa (BN) has common clinical features with attention deficit/hyperactivity disorder (ADHD) and furthermore, that ADHD symptoms are more prevalent in patients with BN [1]–[7]. However, existing evidence is controversial, and little is known about the possible neurocognitive mechanisms that mediate this association.

Patients with BN clinically show increased impulsive behavior such as loss-of-control bingeing and purging [8]. Forty percent of patients with BN also suffer from deficits in impulse control in areas other than eating, such as difficulties in managing negative emotions and sensation seeking [9]. However, the frequency of ADHD-symptoms in patients with BN is not well studied. Moreover, the roles of impulsivity and inattention in the mediation of these symptoms are also unexplored. If BN and ADHD have an additive effect, comorbid patients might show even higher rates of impulsivity and inattention; thus, comorbid patients may have more severe BN symptoms and general psychopathology than patients with BN alone.

Symptoms of BN occur in patients with ADHD with an odds ratio (OR) of up to 3.6 [1]–[4], which demonstrates that the comorbidity of BN among patients with ADHD is strongly elevated. Two studies found an association between childhood impulsivity and the development of BN [3], [4]. Cortese et al. [2] observed a combined association between inattentiveness and impulsivity and a higher likelihood of developing bulimic behaviors.

We identified three studies that examined the prevalence of ADHD in people with eating disorders, including one specific to BN. Wentz et al. [5] found that 9 of 30 (30%) women with different eating disorders had ADHD, although none of these patients was diagnosed with BN. Yates et al. [6] examined 37 female inpatients with BN and 97 female inpatients with binging/purging anorexia nervosa (bpAN) and found that 6.7% of all participants met a diagnosis of childhood-onset ADHD. These authors also found higher current but not childhood inattentive scores measured by the Multi-International Psychiatric Interview (MINI) in patients with BN. Blinder et al. [7] found that 9% of 882 patients with BN also had ADHD; however, this study did not use standardized tools to diagnose BN or ADHD and collected no data on impulsivity, inattentiveness or additional psychopathology. Because only 2.7%–4.8% of women in the general population have ADHD [10], [11], these studies suggest that the prevalence of ADHD symptoms is higher in patients with BN; however, the relative roles of impulsivity and inattention, as well as the impact of comorbidity on psychopathology, remain unclear.

Given that patients with ADHD and those with BN respond to different pharmacological and non-pharmacological treatments, diagnosing comorbidity might lead to new therapeutic opportunities [1]. Patients with a higher body mass index (BMI) are more likely to have ADHD [2] and to develop BN [4] compared with age-matched controls. Early case studies have linked stimulants to weight normalization and fewer binging episodes in patients with BN [12], [13].

We examined patients with BN and healthy controls (HC) to determine the severity of their impulsivity and inattention, as well as the rate of childhood and current ADHD symptoms. Furthermore, we measured bulimic symptoms and general psychopathology in our sample to analyze the potential clinical burden of this comorbidity. We hypothesized that patients with BN would display an increased rate of impulsivity, inattention and comorbid ADHD-symptoms compared with the control group. In addition, we expected current comorbid patients and those with BN who had been diagnosed with ADHD as children to be more impulsive, inattentive and severely affected by bulimia and general psychopathology. Finally, we performed a linear regression analysis to investigate the relative contributions of impulsivity, inattentiveness and hyperactivity to the severity of eating disorder symptoms in order to facilitate the identification of patients at especially high risk.

## Materials and Methods

### Study Sample

We recruited 57 consecutive female patients with BN (age range = 15–35 years, mean = 21.9 years, and standard deviation (SD) = 4.7 years) of average intelligence (mean IQ = 107 and SD = 11.3) and normal body weight (mean BMI = 20.7 kg/m^2^ and SD = 2.7 kg/m^2^) from inpatient and outpatient admissions at three German university hospitals for child and adolescent psychiatry and psychosomatic medicine for adults (Aachen, Berlin and Bochum) and via an advertisement in the outpatient clinic (45 and 12 patients, respectively). None of these patients reported using stimulants or illicit amphetamines at the time of the study. Furthermore, we recruited, via advertisement (without referring to the aims of the study), an age- and IQ-matched control group consisting of 40 women without eating disorders and with normal BMI.

### Ethics Statement

The study was conducted in accordance with the Helsinki Declaration. All patients and legal guardians consented in writing to participate and the local ethics committees “Ethik-Kommission an der Medizinischen Fakultät der Rheinisch-Westfälischen Technischen Hochschule Aachen”, “Ethikkommission der Medizinischen Fakultät der Ruhr-Universität Bochum” and “Ethikkommission der Charité - Universitätsmedizin Berlin” approved the study.

### ADHD

We used one ADHD interview and two ADHD questionnaires to diagnose patients and controls: the Wender-Reimherr Interview (WRI) [14], the ADHD self-rating scale (ADHS-SB) to diagnose current ADHD-symptoms [15] and the Wender Utah Rating Scale (WURS-K) to diagnose childhood ADHD [16].

The WRI is a structured 28-question interview that consists of 7 subscales representing 7 psychopathological domains. The interviewer scores global ratings from 0–2 for each psychopathological domain based on the degree of patient impairment and symptom pervasiveness across situations. A psychopathological domain is rated as above threshold only if both ratings average 1 or higher. ADHD is considered present when the inattention and hyperactivity domain, as well as two or more other domain, are above threshold [15].

The ADHS-SB consists of 22 questions across three subscales that concern current inattention, hyperactivity and impulsivity with intensity ratings ranging from 0–3. The inattentive subtype is present when at least six inattentive items are rated with scores of two or higher. The hyperactive/impulsive subtype is present when at least six hyperactive/impulsive items are rated with scores of two or higher. An ADHD diagnosis of the combined type is present when both of the above criteria are fulfilled [15].

The WURS-K is a 25-item retrospective measure of the ages of 8–10 years. Items are rated from 0–3. When the total score is above 30, there is a 93% sensitivity and 92% specificity for patients to be diagnosed with childhood ADHD [16].

### Neuropsychology

To assess impulsivity and attention more objectively, we applied the TAP (Testbatterie zur Aufmerksamkeitsprüfung) - a comprehensive computer-based neuropsychological battery that consists of tests of go/no-go tasks, divided attention and congruent/incongruent executive functions [17]. The Go/Nogo task required patients to press a key when a symbol signifying a go trial was presented but to withhold from responding when a symbol representing a no-go trial was shown (go/no-go ratio = 20/20).

In the Divided Attention task, the participant is presented with visual and auditory stimuli simultaneously. If visual stimuli on a screen form a square or two identical auditory stimuli follow each other, a button-press is required.

The Compatibility/Non-compatibility test consisted of arrows that pointed left or right and appeared on the left or right side of the screen. The direction of the arrow indicated the hand with which the respective button sh*o*uld be pressed. For example, an arrow appearing on the right side of the screen and pointing to the right was considered compatible and had to be answered by a right hand button press; a response was incompatible if an arrow on the right side of the screen was point*i*ng to the left.

The participants’ reaction times (RTs), the standard deviations (SDs) of RTs and the number of errors or misses committed during the last two subtests were calculated.

### Further Psychopathology

We administered the Eating Disorders Inventory (EDI-II; [18]) and the Structured Interview for Anorexia and Bulimia (SIAB-EX; [19]) to confirm diagnoses and test eating disorder severity. We calculated the total score for both instruments and for the SIAB-EX subscales General Psychopathology, Drive for Thinness, Atypical Bingeing, Lifetime Bulimia and Purging.

We used Beck’s Depression Inventory (BDI-II, [20]), the depression and anxiety subscales of the Symptoms Check List (SCL-90, [21]) and the Structured Clinical Interview for DSM-IV (SKID-I) to further explore comorbid psychiatric disorders, specifically previous lifetime anorexia nervosa (AN) and amphetamine use. The latter two were examined because of the high transition between AN and BN and to examine potential self-medication effects of amphetamine use. Experienced and trained clinicians performed all interviews.

As symptoms of restless legs syndrome can mimic those of ADHD [22], we also administered the Restless Leg Syndrome Rating Scale [23] to control for this potentially confounding diagnosis.

IQ was measured using the Hamburg Wechsler Intelligence Test (HAWIK-IV) for adolescents and the German “Mehrfach Wortschatz Intelligenz-Test B” (MWT-B) for adults.

The 30-item Barratt Impulsiveness Scale (BIS-10) assessed impulsivity [24]. It is composed of three subscales (Attentional/Cognitive, Motor and Non-Planning Impulsivity) and a total score.

### Statistical Analyses

We performed all statistical analyses using SPSS/PASW 18.0.

We compared patients and healthy controls on measures of age, IQ, EDI-II, BDI and SCL-90. Furthermore, we calculated and compared total scores on the WURS-K, ADHD-SB and WRI, as well as the percentage of BN and HC meeting the criteria/cut-offs. We used the two-tailed Student’s t-test for age and IQ and the Mann-Whitney-U-Test for all other analyses to account for non-normal distributions. Alpha was adjusted for multiple testing for each domain separately (ADHD-assessment, neuropsychological assessment, and questionnaires).

Patients only received the full diagnosis of adult ADHD according to the DSM-IV if they met both childhood and current ADHD criteria. We used the most conservative combination for adult ADHD in our sample, requiring the WURS-K (childhood) the WRI and the ADHD-SB (current) to be above threshold to calculate an ADHD and BN comorbidity rate.

Next, we split the BN patients into subgroups: one group with and one group without previous childhood ADHD (WURS-K >30). The patients were placed into subgroups because, as described in the introduction, this condition has been found to increase the risk for the development of an eating disorder [3], [4]. Therefore, previous childhood ADHD, even if it is not any more present as full adult ADHD, can be expected to be important for the underlying neuropsychological processes and/or endophenotypes regarding impulsivity and inattention.

We compared BN-patients with HC and the two subgroups of BN (with former childhood ADHD or without childhood ADHD) using the BIS-10, SIAB-EX, EDI-II, BDI-II, SCL-90 and SCID-I.

We transformed the TAP results for the RTs, the SD of RTs and the misses/errors into age and sex-specific T-values based on German norms because we found the raw values to be age-dependent [20].

To examine if neuropsychological differences between the respective groups are affected by the presence of depressive and anxious symptoms, we also calculated individual linear regressions with the SCL_anxiety and BDI scores to obtain non-linear residuals. Between-group differences in these residuals where then examined by using the Mann-Whitney-U-Tests as described above.

To assess which ADHD symptoms best explained the variance of BN clinical symptoms, a linear regression was performed with the ADHS-SB subscales for impulsivity, inattention and hyperactivity as dependent variables and the SIAB-EX eating pathology total score as independent variable.

## Results

### Sample Characteristics

Patients with BN and HC did not differ with regard to age and IQ; as expected, they did differ significantly with regard to eating disorder characteristics, depression and general psychopathological scores.

### ADHD Prevalence in Patients with BN

In patients with BN, the prevalence of former childhood ADHD (i.e., WURS-K scores greater than 30) was 21.0% (Table 1) versus 2.5% in the HCs (odds ratio, OR: 8.4).

**Table 1:** Study Sample.

	BN (N = 57)				HC (N = 40)					
	Mean		SD		Mean		SD	p		
Age (years)	20.8		4.82		21.2		3.99	n.s.		
IQ	107		11.3		108		14.1	n.s.		
EDI	333		59.6		149		81.5	<0.001		
BDI	23.8		12.9		3.4		5.0	<0.001		
SCL-90	104		64.3		14		17.8	<0.001		
**Childhood ADHD**										
WURS-K (total score)	18.0		15.1		8.2		7.5		0.002	
**Adult ADHD**										
ADHD-SB (total score)	19.4	11.2		5.5		5.7			<0.001	
WRI (total score)	22.6	12.0		12.7		8.9			<0.001	
	% cutoff				% cutoff					
**Childhood ADHD**										
WURS-K	21.0				2.5			0.023		
**Adult ADHD**										
ADHD-SB	24.5				5.0			0.014		
WRI	19.3				5.0			0.048		

Sample characteristics and the results of the instruments characterizing ADHD-symptoms in 57 patients with bulimia nervosa (BN) compared to 40 healthy controls (HC).

HC: Healthy controls, BN: Patients with bulimia nervosa.

For age and IQ, two sided Student’s t-tests were used; for the other analyses, Mann-Whitney-U-Tests were employed due to non-normal distribution. After correction for multiple comparisons, p-values below 0.005 remain significant.

In the ADHD-SB, which measures current symptoms, 24.5% of patients vs. 5.0% of HC scored above the cutoff (OR 5.3). For subtypes, 5.2% of patients belonged to the inattentive subtype, 12.3% of patients were included in the hyperactive/impulsive subtype and 7.0% of patients were included in the combined subtype. In the expert interview, 19.3% of patients vs. 5.0% of HC were rated above the cutoff value (WRI, current symptoms, OR 4.9).

We found that 10.5% of patients scored above the cutoff on all three ADHD measures (the childhood measure and both current ADHD measures) compared to 2.5% of HCs (OR 4.2). The combination of childhood and current ADHD is required to meet a diagnosis of adult ADHD according to the DSM-IV; participants must show continuous symptoms from childhood to adulthood. Fourteen percent of patients showed current symptoms of ADHD without meeting the childhood-onset criteria. Three quarters of these patients had subthreshold childhood symptoms (WURS-K between 15 and 30).

### Neurocognitive Deficits in the BN Group with or without Former Childhood ADHD

This section compares patients with comorbid BN and previous childhood ADHD to those without childhood ADHD. Patients with BN and childhood ADHD did not differ from those without ADHD in age (20.8 vs. 21.2 years, p = n.s., Mann-Whitney-U), IQ (107 vs.108, p = n.s.) or BMI (20.3 vs. 20.69 kg/m2, p = n.s.).

As observed in Table 2, most of the TAP scores were lower than those of HCs; this result suggests that patients with BN might have an impairment in neurocognitive performance. All patients with BN were impaired with respect to the within-subject response variability and number of misses in the Go/no-go task, which are thought to be markers for executive control impairment and impulsivity [33].

**Table 2:** TAP Neuropsychology.

	BN	HC	diff.	BN with	BN w/o	subgroup
				ADHD	ADHD	diff.
	N = 57	N = 40	p	N = 12	N = 45	p
Age	20.8	21.2	n.s.	23.4	20.0	0.021
IQ	107	108	n.s.	109	106	n.s.
GO/NOGO Median RT	49.6	53.0	0.021	46.0	50.6	n.s.
GO/NOGO SD	46.1	55.3	0.001	45.5	46.2	n.s.
GO/NOGO Misses	40.1	54.6	0.001	39.0	40.4	0.054
Vis Divided Attention Med. RT	46.5	47.4	n.s.	43.1	47.4	0.098
Vis Divided Attention SD	50.2	50.9	n.s.	43.1	52.8	0.034
Vis Divided Attention Misses	47.0	46.6	n.s.	43.7	48.2	n.s.
Total Comp/Incomp Median RT	46.8	49.6	0.017	47.8	46.5	n.s.
Total Comp/Incomp SD	46.5	52.1	0.021	42.4	47.6	0.036
Total Comp/Incomp Errors	53.7	51.7	n.s.	50.1	54.6	n.s.

T-values from all patients with BN compared with HCs and from patients with BN and childhood ADHD compared to patients without comorbid ADHD are shown below.

A Whitney-Mann-U test was used to test for significant differences between BN and HC and between subgroups split by childhood (WURS-K) ADHD diagnosis. After correction for multiple comparisons, p-values below 0.005 remain significant.

BN with ADHD: Bulimia nervosa patients without childhood-onset ADHD.

BN w/o ADHD: Bulimia nervosa patients with childhood-onset ADHD.

RT = reaction time.

SD = standard deviation.

In general, patients with BN and childhood ADHD had a tendency towards lower neuropsychological test scores than those without childhood ADHD indicating more inattentive and impulsive response patterns. Specifically, patients with BN and childhood ADHD showed inconsistent and variable responses in the Divided Attention and Incompatibility task. However, these results did not survive a correction for multiple comparisons.

Table 3 displays the self-rated impulsivity results (BIS-10). Patients were uniformly and significantly more impaired than HCs. Patients with comorbid BN and childhood ADHD were more severely affected than those with BN alone with regard to both the total BIS and the Attentional/Cognitive subscale scores and showed a trend towards significance in the BIS-Motor subscale.

**Table 3:** Impulsivity.

	BN	HC	diff.	BN with	BN w/o	subgroup
				ADHD	ADHD	diff.
	N = 57	N = 40	p	N = 12	N = 45	p
BISNon-Planning	23.5	19.1	<0.001	24.7	23.2	n.s.
BISMotor	24.7	12.8	<0.001	27.8	23.7	0.052
BISAttentional/Cognitive	28.1	16.7	<0.001	30.5	27.3	0.007
BISTotal	70.6	48.5	<0.001	79.3	68.1	0.011

Data from all patients with BN compared to controls and patients with BN and childhood ADHD compared to patients without comorbid ADHD are shown below.

A Mann-Whitney U Test was used to test for significant differences. After correction for multiple comparisons, p-values below 0.016 remain significant.

BN with ADHD: Bulimia nervosa patients without childhood-onset ADHD.

BN w/o ADHD: Bulimia nervosa patients with childhood-onset ADHD.

### Severity of Illness in Patients with Comorbid BN and Childhood ADHD in Comparison to those without Childhood ADHD

Table 4 displays the results of disordered eating and further psychiatric symptom assessments. As expected, the EDI-II and SIAB-EX total scores were in the pathological range for all patients with BN. All patients with BN also showed more anxiety and depression as measured by the SCL-90 and the BDI-II, respectively, compared with HCs. Patients also showed a higher lifetime prevalence of amphetamine consumption and a higher rate of lifetime anorexia nervosa (AN) diagnoses compared with controls. The largest group differences between patients with comorbid BN and ADHD and those without ADHD were found in the SIAB-EX total score (effect size η^2^ = 0.14) as well as the anxiety subscale of the SCL-90 (η^2^ = 0.24). Although patients comorbid with BN and ADHD had a tendency to be more severely affected across most measures, this result did not hold for lifetime AN, which demonstrated an opposite trend.

**Table 4:** Clinical scales.

	BN	HC	diff.	BN with	BN w/o	subgroup
				ADHD	ADHD	diff.
	N = 57	N = 40		N = 12	N = 45	p
SIAB-EX	93.0	n.a.	n.a.	116.3	86.5	0.005
EDI-II	336	149	<0.001	380	326	0.024
SCL-90 total	104	14	<0.001	155	89.2	0.008
SCL-90-depression	21.0	2.8	<0.001	28.8	18.5	0.010
SCL-90-anxiety	10.2	1.2	<0.001	17.8	8.1	0.003
BDI-II	23.8	3.4	<0.001	28.5	22.4	n.s.
RLS sum	4.86	n.a.	n.a.	5.17	4.78	n.s.
Amphetaminelifeti. (%)	12.3	0	0.002	25	8.8	n.s.
AN lifetime (%)	46.6	0.25	<0.001	11.6	37.7	0.057

Data from all patients with BN compared to controls and patients with BN and childhood ADHD compared to patients without comorbid ADHD are shown below. The average values of SCL-90, BDI-II, SKID-I and SIAB-EX eating pathology measures for all patients with BN (BN), healthy controls (HC) as well as subgroups split by WURS-K ADHD diagnosis are shown above. The Mann-Whitney U test was used to examine significant differences. After correction for multiple comparisons, p-values below 0.006 remain significant.

BN with ADHD: Bulimia nervosa patients without childhood-onset ADHD.

BN w/o ADHD: Bulimia nervosa patients with childhood-onset ADHD.

n.a. = not available.

We found no correlations among psychiatric symptoms and age, IQ or BMI. Inpatients did not score higher on comorbidity or any other psychiatric symptoms compared with outpatients (data not shown).

After controlling for the effects of depressive and anxious symptoms for BN vs. HCs, the following group differences remained significant: BIS scales, TAP Go/Nogo standard deviation, EDI total score and SCL-total score; thus, these variables differ between BN and HCs independent from depression and anxiety. For the comparison between BN with and without childhood-onset ADHD, only the TAP Visual Divided Attention standard deviation and Total Compatible/Incompatible standard deviation remained significant; BIS-total score (p = 0.095) and EDI total score (p = 0.090) showed trends towards significance.

### The Relative Influence of Impulsivity, Inattention and Hyperactivity

Patients with comorbid BN and childhood ADHD scored significantly higher on the ADHD-SB subscale for current inattention than those without childhood ADHD (13.42 vs. 8.88, Mann-Whitney U, p<0.019). There was a trend towards more current hyperactivity (7.33 vs. 4.50 p = 0.063) and current impulsivity (5.67 vs. 3.68, p = 0.237).

When examining which of the ADHD subscales was responsible for most of the variance of eating disorder symptoms, we found that inattention explained more of the variance of eating disorder symptoms than either impulsivity or hyperactivity. Taken together, these subscales explained 36% of the variance of the SIAB-EX eating disorder total score (linear regression, F = 7.50 p<0.001; β_inattention_ = 0.323, p<0.001; β_hyperactivity_ = 0.199, p = 0.261; and β_impulsivity_ = 0.170, p = 0.330). Including impulsivity as measured by the BIS-10 of the model did not significantly increase the total explained variance (39%); moreover, the BIS-10 did not significantly predict eating disorder symptoms (β_BISimpulsivity_ = −0.208, p = 0.147). Similar results were obtained when using the EDI-II as a dependent variable (data not shown).

## Discussion

In our study patients with BN were more impulsive and more inattentive than HCs. Twenty-one percent of BN-patients met the criteria for former childhood ADHD vs. 2.5% in HCs. Patients with childhood ADHD showed more impulsivity, inattention and eating disorder symptoms. Inattentive, rather than impulsive, patients with BN had the greatest risk to also have ADHD. In our sample, 10.5% of the 57 patients with BN currently had full ADHD according to the DSM-IV (i.e., childhood and both current ADHD-instruments scored above cut-off). This rate is much higher than the 2.5% rate found in our control sample, the 2.7% rate found in females worldwide [10] or the 4.8% rate found in a German population using the WURS-K and ADHD-SB but not the WRI [11].

These results fit published results by Yilmaz et al. [25] who recently conducted a retrospective study of patients with BN recruited via advertisement and found a similarly elevated rate of childhood ADHD in patients with BN (23.5%). The only previous study to have examined adult ADHD in BN found that 9% of inpatients were comorbid [7]. The comorbidity OR of 4.2 in our study is similar to previous inverse studies of BN in ADHD (OR = 3.6; [1], [3], [4]). Together, these findings suggest that the rate of ADHD is significantly higher in treatment-seeking patients with BN than in HCs.

We examined the subgroup of patients with childhood ADHD because Mikami [3], [4] found that childhood/adolescent ADHD was a significant risk factor for BN in adulthood. We found differences in the subjective and objective measures of impulsivity and attention between patients with BN and childhood ADHD compared with those with BN alone. The BIS-10 revealed significantly more impulsivity (BIS-total = 70.6) in all patients with BN compared with HCs (48.5, p<0.001) [26]; this result is in line with previous findings of higher impulsivity, negative urgency, lack of planning and sensation-seeking in patients with BN [27]. Patients with BN and childhood ADHD were even more impulsive than those with BN alone, as measured by the total BIS score, which suggests that there is an additive effect with regard to impulsiveness. It is noteworthy that the BIS subscale “Attentional Impulsivity”, which describes impulsive and inattentive behavior, was also significantly more impaired for patients with BN and childhood ADHD. Patients with ADHD also scored high on this subscale in other examinations [26], potentially because it captures two key symptoms of ADHD (i.e., impulsive and inattentive behavior). Patients with BN and childhood ADHD also scored significantly higher on the ADHS-SB subscale for inattentiveness in adulthood and showed a trend toward higher hyperactivity compared with patients without childhood ADHD.

Our study also confirms more impulsive and inconsistent response patterns for treatment-seeking patients with BN in the neuropsychological test battery. Some previous neuropsychological studies on these patients showed similar impulsive behaviors [28], [29]. However, previous findings were mixed and conclusions unclear [30], [31]; thus our study supports further evidence of neuropsychological deficits in BN. These neuropsychological deficits had a tendency to be even more pronounced in patients with BN and childhood ADHD, especially impulsive response patterns and attentional problems. Again, this tendency towards a higher intensity of neuropsychological abnormalities in comorbid patients could suggest an additive effect of ADHD and BN with regard to impulsivity and inattention.

In addition, we found markedly more eating behavior pathologies in patients with BN and childhood ADHD based on the SIAB-EX total scores compared with patients without ADHD. Patients with BN and ADHD also had significantly more severe psychiatric symptoms (SCL-90 total score) and scored higher on the SCL-90 subscales of depression and anxiety but not amphetamine use. These symptoms are typically found in BN [7]; however, the occurrence of the aforementioned symptoms was significantly more frequent in comorbid patients compared with those with BN alone.

We questioned whether the above effects were due to the greater general psychopathological burden of patients with two diagnoses or whether there is a specific interaction between BN and ADHD. Virtually any illness combination might pose a risk of increased psychosocial stress and additional clinical complications compared with only one diagnosis [32]. Depressive and anxious symptoms in our sample, which were quite prevalent, have the potential to interact with the effects of additional ADHD symptoms and could be responsible for some of the findings. The fact that not all differences remained significant after correcting for the effects of depressive and anxious symptoms supports this hypothesis. However, a closer inspection of these data suggests that there is a more complex interaction between BN and ADHD. Not all of the symptoms that we assessed were uniformly more severe, as would be expected from a general effect alone. With regard to lifetime AN diagnosis, we even detected the opposite trend: lifetime AN tended to be more common in patients with BN alone compared with those who have comorbid BN and childhood ADHD. In addition, according to the literature, the rate of comorbid disorders in patients with ADHD is not homogeneous: conduct disorder (OR = 10.7), borderline personality disorder (OR = 5.6), depression (OR = 5.5) and anxiety (OR = 3.0; [27], [32]) differ significantly in their prevalence, which indicates complex interactions among certain comorbidities. Also, key differences between the groups, such as the increased standard deviation on the TAP neuropsychological examination, remained significant after correcting for depressive and anxious symptoms, and increased impulsivity and disordered eating symptoms still showed a trend towards significance. This could not be explained by a general effect of depressive or anxious symptoms alone. Furthermore, Fayyad et al. [10] showed that the occurrence of ADHD generally predated the development of depression or anxiety. This points toward at least partly independent effects of childhood ADHD. Taken together, the high symptom rate for patients with BN and ADHD likely represents the combination of a general effect as well as a more specific pathophysiological interaction.

We analyzed the relative impact of impulsivity, inattention and hyperactivity on the severity of eating disorder symptoms. Inattention in adulthood explained most of the variance within the eating pathology for patients with BN. This study is the first to directly show this relationship. Cortese et al. [2] found indirect evidence that adolescent inattention and impulsivity (but not hyperactivity) together predicted bulimic behavior five years after ADHD-diagnosis. Moreover, Altfas et al. [33] found that obese patients with ADHD who were at risk for eating disorders were predominantly inattentive. In addition, Yates et al. [6] found inattentiveness and hyperactivity symptoms in adults with either AN or BN. These findings are consistent with our results. Furthermore, several authors have found a potential relationship between impulsivity and BN: Mikami et al. [3], [4] found that childhood impulsiveness predicted adolescent BN symptoms 5–8 years after ADHD-diagnosis, and Wonderlich et al. [34] found that impulsivity increases vulnerability for BN in general. Thus, childhood impulsivity is also an important risk factor for BN. The same result might be true for adult impulsivity, as we found a considerably higher rate in our sample. However, whether this correlation also has causal effects can only be answered in a longitudinal examination.

In summary, we know from previous studies that childhood impulsivity is a significant risk factor for developing BN in adolescence or early adulthood. Our study showed that patients with BN who are more impulsive than average and those who are also more inattentive may be at an even higher risk for severe eating disorders.

There are several possible ways in which ADHD and BN might interact. ADHD impulsiveness likely contributes to binge eating and purging [2], [27]; however, this is only one possibility because we found only a weak correlation between impulsivity measures and BN symptoms within our participants. Additionally, binge eating/purging subscales were not correlated with impulsivity measures (data not shown). This finding matches previous studies that did not find correlations between binge eating frequency and impulsivity [35]. However, inattentiveness might contribute to BN behavior. For example, binge eating may act as a compensatory mechanism to help control the frustration associated with the attentional and organizational difficulties typical in ADHD [36], [37]. Furthermore, attention deficits might cause people to ignore hunger, satiety or both. Studies in our lab found evidence for this hypothesis: patients with ADHD were more likely to eat more and to be significantly overweight compared with healthy controls [38]. Parental ratings of inattention and neuropsychological measures of divided attention predicted the amount of readily available snacks eaten in a standardized setting [39]. Also, a representative nationwide German study linked hyperactivity/inattention with increased food intake in healthy adolescents [40] and hyperactivity/inattention was also related to inability to lose weight in obese patients with ADHD [41]. Furthermore, genetic mechanisms and differences in neurobiological functioning in patients with ADHD have been linked to overeating [36], [40]. Lastly, part of the interaction might involve increased levels of depression and anxiety which are known comorbidities for both BN and ADHD. The occurrence of depression and anxiety could cause more emotional stress, which would worsen both ADHD and BN symptoms.

### Limitations

Because our study was cross-sectional, we cannot make causal or developmental inferences. In addition, the age range sampled does not allow us to generalize our results to adolescents or adults alone; however, we sampled the most common onset age of BN, which presents an ecologically valid representation of treatment-seeking patients with BN. Treatment-seeking might lead to a higher rate of comorbidity than has been found in population-based samples. To control for this effect, an additional control group, consisting of non-treatment seeking patients with BN would be needed. Within our group, however, inpatients did not report more comorbidities or stronger psychopathologies than outpatients, so there seems to be no overt effect of treatment modality. Nevertheless, the results of our study should only be generalized to treatment-seeking patients with BN. Furthermore, our findings may in part reflect an interaction effect with the consequences of possible substance abuse, which is quite common in BN [9]. Prolonged substance abuse may cause inattention, which would not be a comorbidity of BN and ADHS [25], [41]. However, as we used ADHD in childhood to separate the subgroups, long-standing substance abuse seems less likely as a primary cause; it might, however, contribute to symptom severity. Lastly, prolonged starvation might also be a cause for inattention problems [6]. However, in our sample, BN patients with a history of AN had a tendency to occur more frequently in the subgroup without ADHD than in the subgroup with ADHD.

The strength of this study is that it is a multidimensional assessment. We used several measures of ADHD, impulsivity, inattention, eating disorders and general psychopathology simultaneously. In addition, we combined self-report questionnaires with expert interviews and objective neuropsychological tests. Thus, we obtained valid results for each category to calculate group comparisons and cross-correlations.

Because of the high comorbidity between BN and ADHD, as well as the higher burden for patients with this comorbid condition, clinicians should screen patients with BN for ADHD. A combination of self-report questionnaires and semi-structured interviews can diagnose ADHD in patients with BN. Neuropsychological tests can also support the diagnosis. Care should be taken to test for childhood ADHD symptoms to obtain a complete DSM-IV diagnosis; however, the assessment of symptoms that appear before the age of 12 might suffice [42]. The pharmaceutical and non-pharmaceutical treatment options that are commonly applied to ADHD might be helpful for patients with comorbid ADHD and BN. Early case reports have demonstrated that stimulants caused less bingeing and purging in these patients who were able to maintain a normal weight [12], [13], [21], [22]. Studies such as these are important steps toward finding an adequate treatment for BN.
